# Estrogen deficiency – a central paradigm in age-related impaired healing?

**DOI:** 10.17179/excli2020-3210

**Published:** 2021-01-11

**Authors:** Mohamed El Mohtadi, Kathryn Whitehead, Nina Dempsey-Hibbert, Amina Belboul, Jason Ashworth

**Affiliations:** 1 Department of Biology, Edge Hill University, Ormskirk, Lancashire, L39 4QP, UK; 2 Centre for Bioscience, Manchester Metropolitan University, Chester Street, Manchester, M1 5GD, UK

**Keywords:** wound healing, aging, age-related impaired healing, estrogen, estrogen receptors

## Abstract

Wound healing is a dynamic biological process achieved through four sequential, overlapping phases; hemostasis, inflammation, tissue proliferation and remodeling. For effective wound healing, all four phases must occur in the appropriate order and time frame. It is well accepted that the wound healing process becomes disrupted in the elderly, increasing the propensity of non-healing wound states that can lead to substantial patient morbidity and an enormous financial burden on healthcare systems. Estrogen deprivation in the elderly has been identified as the key driver of age-related delayed wound healing in both genders, with topical and systemic estrogen replacement reversing the detrimental effects of aging on wound repair. Evidence suggests estrogen deprivation may contribute to the development of chronic wound healing states in the elderly but research in this area is somewhat limited, warranting further investigations. Moreover, although the beneficial effects of estrogen on cutaneous healing have been widely explored, the development of estrogen-based treatments to enhance wound repair in the elderly have yet to be widely exploited. This review explores the critical role of estrogen in reversing age-related impaired healing and evaluates the prospect of developing more focused novel therapeutic strategies that enhance wound repair in the elderly via activation of specific estrogen signaling pathways in regenerating tissues, whilst leaving non-target tissues largely unaffected.

## Background

Declining levels of estrogen in both genders with increasing age suggests that age-related impaired wound healing may result in part from the loss of protection that was once afforded by estrogen during youth. Indeed, estrogen treatments appear to reverse the detrimental effects of age-related impaired healing, resulting in accelerated wound repair in both genders. Despite these findings, the use of estrogen-based treatments to reverse delayed healing in the elderly has not been widely adopted outside research settings. Moreover, the potential role of the sex steroid hormones in chronic wounds remains unclear but evidence suggests that being male is a risk factor for venous ulceration, whilst the use of hormone replacement therapy (HRT) by post-menopausal women appears to reduce the risk of venous ulceration (Bérard et al., 2001[22]; Margolis et al., 2002[82]). Furthermore, polymorphisms in the estrogen receptor-beta (ER-β) gene are associated with venous ulceration (Ashworth et al., 2005[16], 2008[15]). Thus, it is feasible that estrogen deprivation may contribute to the development of chronic wound healing states in the elderly. The lack of extensive research in this area highlights the need for further investigations to explore the precise mechanisms by which estrogen deficiency may contribute to the development or progression of chronic wounds in the elderly. This review explores current knowledge in this field, highlighting the critical role of estrogen in reversing age-related impaired healing and prospects for developing more focused therapies in the form of local dressings that promote healing in the elderly via activation of specific estrogen signaling pathways in regenerating tissues, whilst leaving other non-target tissues in the body largely unaffected.

## Acute Wound Healing

**Figure 1:**
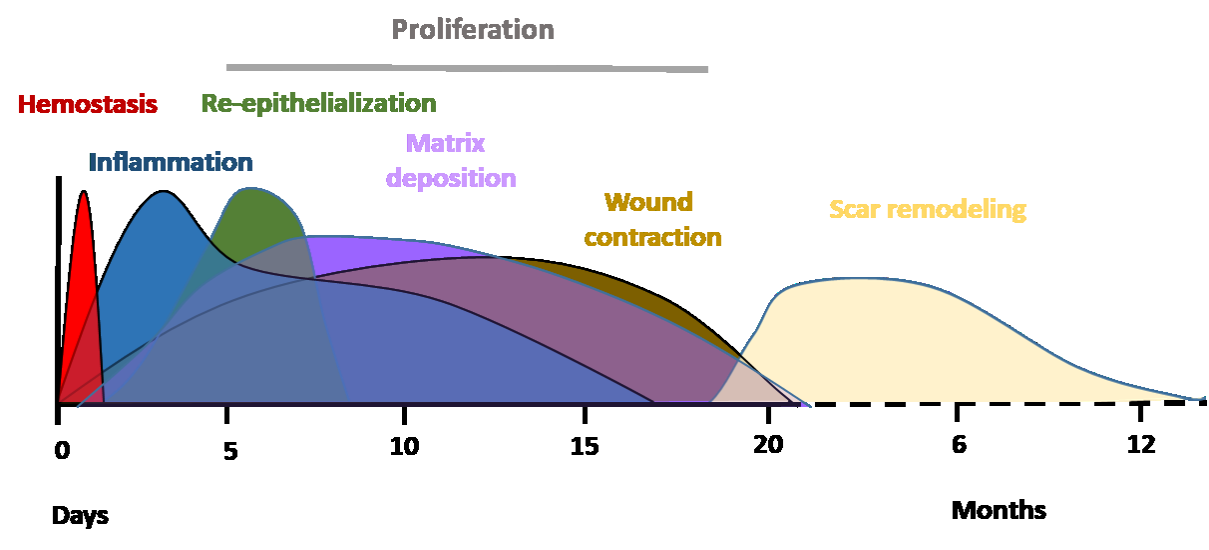
Typical timescale and phases of acute wound healing stages in young adult humans. Immediately after injury, healing initiates with hemostasis. This results in the formation of a fibrin clot within minutes following injury. The inflammatory phase overlaps with hemostasis and occurs within minutes after injury, with neutrophils being recruited from circulation, followed by monocytes. Monocytes undergo a series of changes to differentiate into tissue macrophages, which carry out phagocytosis and release cytokines that encourage the recruitment and activation of further leukocytes to the injury site and initiation of the proliferation phase. Three to ten days after injury, the proliferation phase starts enabling granulation tissue formation, re-epithelialization and angiogenesis. The final phase is the remodeling of a mature tissue scar, which can take several months or, in some cases, up to a year post-injury. 0 = day of wounding/injury (El Mohtadi, 2019).

Acute wound healing is a complex and dynamic biological process divided into four sequential, overlapping phases; hemostasis, inflammation, tissue proliferation and remodeling of the tissue scar (Figure 1(Fig. 1); Reference in Figure 1: El Mohtadi, 2019[41]). Immediately after trauma, degranulating platelets adhere to damaged blood vessels and start a hemostatic reaction, increasing the coagulation cascade and producing a fibrin clot to prevent extreme blood loss and provide a temporary protection for the wound against foreign bodies (Vaughan et al., 2000[147]; Weyrich and Zimmerman, 2004[152]; Gilliver et al., 2007[50]). Platelets in the clot release a variety of pro-inflammatory cytokines and growth factors including platelet-derived growth factor (PDGF), transforming growth factor-beta (TGF-β), fibroblast growth factor-2 (FGF-2), vascular endothelial growth factor (VEGF) and epidermal growth factor (EGF) (Bauer et al., 1985[19]; Guo and DiPietro, 2010[54]). These cytokines, chemokines and growth factors attract inflammatory cells from circulation to the wound site, initiating the inflammatory phase. Neutrophils are the first inflammatory cells recruited from circulation (Ley et al., 2007[77]). They peak in numbers at 24 to 36 hours post-injury (Dovi et al., 2004[39]). Neutrophils remove foreign materials and invading microorganisms, such as bacteria, via the release of reactive oxygen species (ROS) and lysosomal enzymes, and degrade damaged matrix tissues by collagenases and proteinases (Mosser and Edwards, 2010[95]). The majority of neutrophils are enclosed in the wound clot and are either eliminated with the eschar or by macrophages via phagocytosis (Newman et al., 1982[100]; Kondo and Ishida, 2010[70]).

In response to chemoattractants such as TGF-β, macrophage chemoattractant protein 1 (MCP-1), and macrophage inflammatory protein (MIP), monocytes from the bloodstream subsequently arrive at the wound area and differentiate into tissue macrophages, peaking in numbers around day 5 to day 7 post-injury (Lorenz and Longaker, 2008[79]; Sen and Roy, 2008[125]). Macrophages replace neutrophils as the predominant inflammatory cells at the wound site and carry out the process of phagocytosis of invading microorganisms, removal of damaged tissues and dead neutrophils, and the release of growth factors such as PDGF and TGF-β (Beanes et al., 2003[20]; El Mohtadi et al., 2020[42]). Damaged extracellular matrix is degraded by the action of macrophage-derived proteolytic enzymes such as metalloproteases. Macrophages also release growth factors that induce the proliferative phase including insulin-like growth factor-1 (IGF-1), keratinocyte growth factor (KGF), epidermal growth factor (EGF) and vascular endothelial growth factor (VEGF) (Shaw et al., 1990[128]). Three to ten days after injury, tissue proliferation starts. It is characterized by the creation of new extracellular matrix (ECM) by fibroblasts, re-epithelialization (the restoration of an intact epidermis) by keratinocytes and angiogenesis (revascularization) by endothelial cells. The final phase is remodeling of the tissue scar, which can take several months or, in some cases, up to a year post-injury. It is characterized by the remodeling of collagen and the vascular maturation of newly formed capillaries, allowing vascular density to return to normal within the wound (Guo and DiPietro, 2010[54]). For successful healing, wound repair requires progression through all four phases in the correct order and time frame (Singer and Clark, 1999[130]; Guo and DiPietro, 2010[54]).

## Aging and Wound Healing

**Figure 2:**
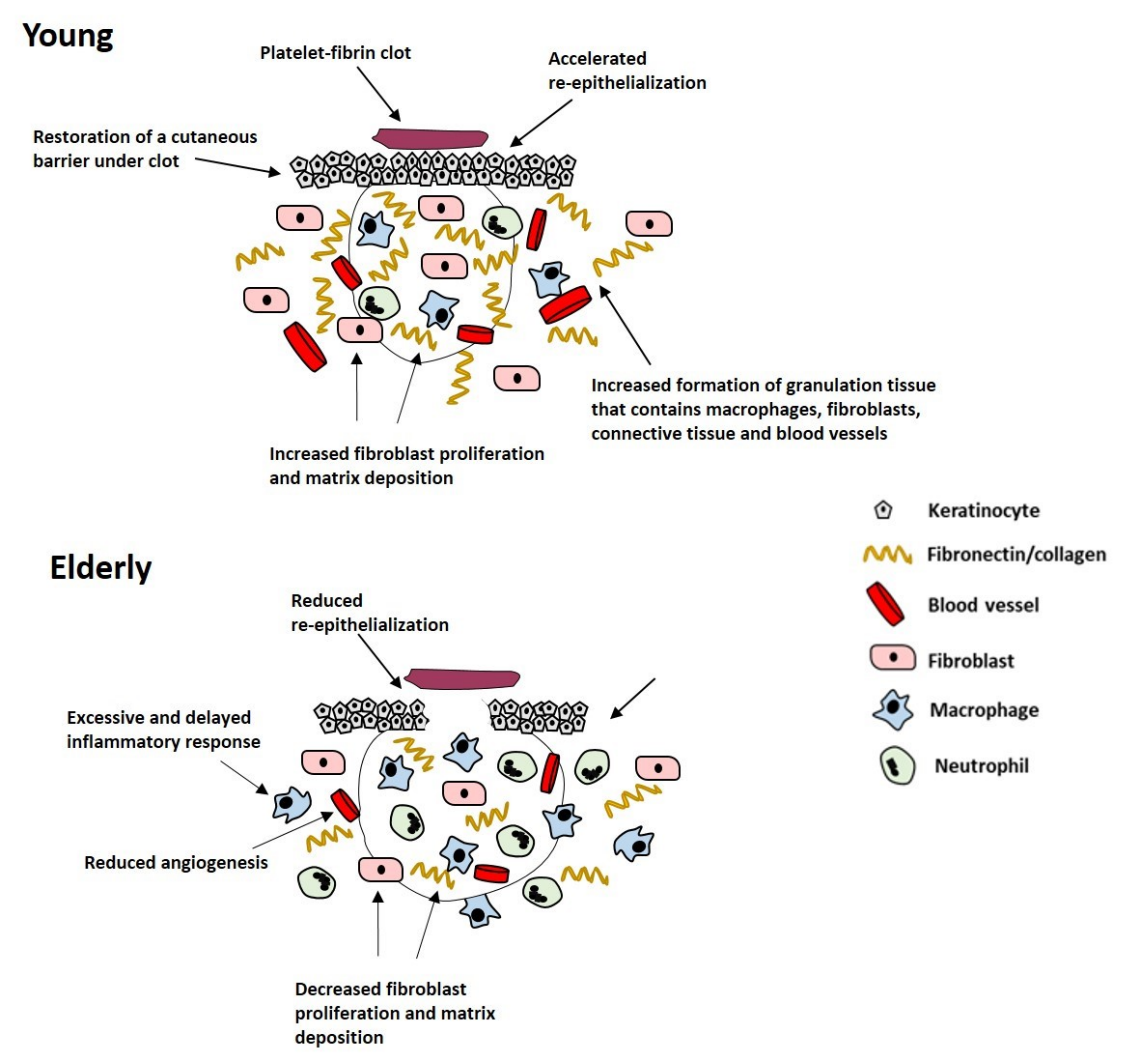
Schematic representation of the effect of age on acute wound healing. Age-related impaired healing is linked to delayed but excessive inflammation, delayed re-epithelialization, reduced angiogenesis and decreased fibroblast proliferation and matrix deposition (El Mohtadi, 2019).

With increasing age, acute wound healing proceeds but becomes delayed. This detrimental change in acute wound healing in the elderly is called age-related impaired healing and is linked with intrinsic cellular aging processes, including an elevated but delayed inflammatory response, reduced cell proliferation and migration, decreased extracellular matrix (ECM) production and increased enzymatic degradation of tissues leading to skin fragility (Thomas, 2001[144]). Delayed wound healing in the elderly is associated with delayed hemostasis (Ashcroft et al., 1999[9]), prolonged and excessive inflammation, delayed re-epithelialization, impaired angiogenesis and reduced matrix deposition (Figure 2(Fig. 2); Reference in Figure 2: El Mohtadi, 2019[41]) (Ashcroft et al., 1997[8], 2002[10]). Although the inflammatory response becomes more pronounced with increasing age, the propensity for wounds to become infected increases in the elderly (Ashcroft et al., 2002[10]; Cooper et al., 2015[37]), due to the delay in wound repair and the impaired ability of inflammatory cells to eliminate bacteria from the wound site (Emori et al., 1991[46]; Thomas, 2001[144]).

In contrast, chronic wounds are characterized by failure of tissue repair processes to proceed through an orderly set of wound healing phases within an expected time frame. Wounds are deemed chronic if they do not heal within three months and in many cases they can take several months or even years to heal (if they heal at all) (Mustoe, 2005[97]; Adeyi et al., 2009[1]). Chronic wounds typically affect the elderly (over 65 years of age) and arise from one or more underlying pathologies, with more than 90 % of chronic wounds being venous, diabetic or pressure ulcers (Boulton et al., 2005[24]). Chronic wounds have major clinical implications and cause an enormous burden on healthcare services, in terms of medical effort and cost (Harding et al., 2002[55]; Boulton et al., 2005[24]). Chronic wound treatment costs the UK National Health Service (NHS) about £5 billion per annum (Guest et al., 2015[52]). 

At present, effective therapies/treatments for chronic wounds are somewhat limited, making this an area of research that needs urgent attention. Chronic wounds become trapped within the inflammatory phase of wound repair and are characterized by an excessive, unabated inflammatory response that leads to tissue breakdown (Snyder, 2005[133]; Taylor et al., 2005[143]; Fazli et al., 2009[47]). A shift in the balance between the formation and degradation of ECM occurs, leading to ECM breakdown by destructive inflammatory mediators such as proteases (Edwards et al., 2004[40]; Schönfelder et al., 2005[124]). Chronic wounds also have defective macrophage function that leads to increased propensity of bacterial infection, decreased growth factor secretion, impaired angiogenesis and delayed re-epithelialization (Hohn et al., 1976[60]; Harding et al., 2002[55]; Frykberg and Banks, 2015[48]).

## Estrogen and Aging

Endogenous estrogens are produced from cholesterol, initially by several enzymes to create androgens, such as testosterone and androstenedione, which are then converted to estrogens through the action of the P450 enzyme aromatase, in the endoplasmic reticulum of estrogen-producing cells (Payne and Hales, 2004[106]). In adipose tissues, androstenedione is converted to estrone whilst in ovarian granulosa cells testosterone is converted into estradiol. Aromatase is found in many peripheral tissues such as skin, bone, adipose tissue, brain and vascular smooth muscle (Nawata et al., 1995[98]; Simpson, 2000[129]; Azcoitia et al., 2001[17]; Ling et al., 2004[78]). In females at the age of reproduction, systemic estrogen is produced mainly by the ovary. It is predominantly biosynthesized in granulose cells of the ovarian follicles and the corpora lutea. In males, the gonad is the principle producer of systemic estrogen. However, a substantial amount of estrogen is also produced locally in peripheral tissues in both genders, acting in an autocrine and paracrine manner (Labrie et al., 1998[73]). A significant amount of inactive steroid precursors including dehydroepiandrosterone (DHEA), its sulphate (DHEA-S), and androstenedione (4-dione) are produced by the adrenals and converted into active steroid hormones in peripheral tissues (Labrie et al., 1998[73]). Several peripheral human tissues, such as adipose tissue, bone and skin can produce active estrogens and androgens locally from conversion of adrenal-derived inactive precursors (Nelson and Bulun, 2001[99]). Plasma DHEA-S is the major adrenal-derived steroid precursor and levels in adult men and women are around 100 to 500 times higher than those of testosterone and as much as 1000 to 10 000 times higher than those of estradiol (Labrie et al., 2000[74]). Thus, inactive adrenal-derived steroid precursors provide a large circulating reservoir for conversion into potent sex steroid hormones in peripheral tissues. However, the sharp decline in DHEA and DHEA-S production by the adrenals during aging in both genders results in a dramatic fall in the synthesis of active androgens and estrogens in peripheral tissues, a phenomenon which could be associated with several age-related diseases (Labrie et al., 1998[73]). Estrogen synthesized locally in peripheral tissues becomes progressively more important after the menopause in women, when systemic levels are lost (Picard et al., 2000[109]). However, the rapid decline in local production of active estrogens with increasing age means peripheral estrogen production is insufficient to compensate for the loss in systemic estrogen levels in elderly women.

## Estrogen Receptors

Over the past decades, the existence of two nuclear estrogen receptor (ER) proteins have been identified, ER-alpha (ER-α) and ER-beta (ER-β), that are part of the nuclear receptor (NR) family. ER-α was first discovered in 1958 (Jensen and Jacobson, 1960[65]) and is known to be predominant in reproductive tissues (Kuiper et al., 1997[71]; Ali and Coombes, 2000[4]; Campbell et al., 2010[33]) whereas ER-β was first identified in rat prostate and ovary in 1996 (Mosselman et al., 1996[94]) and predominates in peripheral, non-reproductive tissues (Kuiper et al., 1997[71]; Ali and Coombes, 2000[4]; Campbell et al., 2010[33]). The biological effects of estrogens are largely mediated by the binding of estrogen to nuclear ER homodimers or heterodimers (Matthews and Gustafsson, 2003[84]), and subsequent activation or repression of gene transcription (Paige et al., 1999[103]). However, rapid, non-genomic estrogen signaling involving membrane-bound ER proteins has also been described (Gruber et al., 2002[51]; Ascenzi et al., 2006[6]). Recent research suggests estrogen can have direct effects on inflammatory cells, such as monocytes and macrophages, and skin-associated cells such as keratinocytes, due to the presence of nuclear and membrane-bound ER proteins (Weusten et al., 1986[151]; Stimson, 1988[137]; Cocchiara et al., 1990[36]). The response of particular inflammatory cells depends on the local levels of estrogen and the maturity (stage of differentiation) of the cells (Ashcroft and Ashworth, 2003[7]).

**Figure 3:**
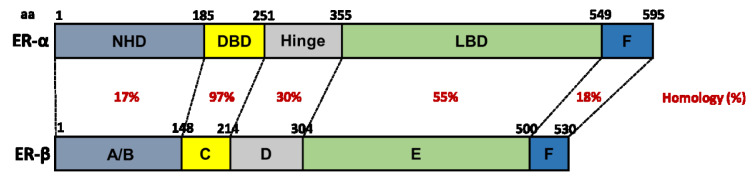
The structure of human ER-α and ER-β. Homology between domains (A-F) is represented as percentage (%) similarity. NHD = N-terminal homology domain, DBD = DNA-binding domain, LBD = ligand-binding domain (Webb et al., 1999; Klinge, 2000; Begam et al., 2017; El Mohtadi, 2019)

Estrogen signals predominantly by binding to inactive ER proteins in the nucleus of the cell (Klinge, 2000[69]). ER proteins share a structure (Figure 3(Fig. 3); References in Figure 3: Webb et al., 1999[149]; Klinge, 2000[69]; Begam et al., 2017[21]; El Mohtadi, 2019[41]) that is typical of the NR family, consisting of six domains (*A-F*) (Kuiper et al., 1998[72]; Klinge, 2000[69]; Begam et al., 2017[21]). ER proteins are expressed in skin, suggesting estrogen regulates skin function, maintenance and/or turnover (Ashworth, 2005[14]). While ER-α and ER-β have 97 % homology in the *C domain* that acts as a DNA-binding domain (DBD), they only have 55 % homology in the *E domain* which forms the ligand-binding domain (LBD) (Barkhem et al., 1998[18]; Webb et al., 1999[149]; Klinge, 2000[69]), enabling targeted ER activation using artificial ligands with ER-specific binding affinity.

When estrogen binds to ER proteins, they become activated and dimerize (Klinge, 2000[69]). The DBD of each activated ER then binds to an estrogen response element (ERE) in the DNA of target genes and induces gene transcription (Kuiper et al., 1998[72]; Klinge, 2000[69]). In cells expressing a single ER subtype, homodimers of ER-α or ER-β are formed (Kuiper et al., 1998[72]). In cells that express both ER subtypes, a heterodimer containing one ER-α and one ER-β may form (Kuiper et al., 1998[72]). ER heterodimers and ER-α homodimers bind to DNA with a similar affinity. However, ER-β homodimers bind to DNA with a lower affinity (Kuiper et al., 1998[72]).

Both ER-α and ER-β enhance aspects of acute wound repair but their roles are somewhat different; although ER-α regulates inflammatory cell activity, ER-β appears to modulate the overall wound healing response (Emmerson and Hardman, 2012[45]). The delayed wound repair observed in ovariectomized mice can be reversed by stimulation of ER-β alone, whilst ER-α activation alone fails to enhance murine wound repair (Campbell et al., 2010[33]). Moreover, estrogen replacement therapy in ovariectomized mice lacking functional ER-β retards wound healing, suggesting ER-β may be critical to establishing prompt tissue formation during wound repair (Campbell et al., 2010[33]). In addition, a human study conducted by Ashworth (2005[14]) indicates that polymorphisms in the *0N* promoter region of the human ER-β gene are significantly associated with chronic venous ulceration in the British Caucasian population.

## Effect of Estrogen on Skin Maintenance

It is commonly accepted that the age-related reduction in estrogen levels is linked with skin degeneration. However, most evidence in humans comes from studies performed in pre- and/or post-menopausal women. During pregnancy, skin syndromes such as psoriasis have been shown to improve, an effect that is directly linked to increased estrogen levels in the circulation (Boyd et al., 1996[25]). Moreover, oral contraceptive pills are frequently used to treat severe acne. During the menopause, estrogen deficiency results in detrimental changes in skin appearance including sagging, wrinkling, dryness and fragility (Ashcroft et al., 1999[9]; Shah and Maibach, 2001[127]). These changes can often be reversed during the first 6 months of topical or systemic estrogen replacement therapy (Brincat et al., 1987[27]).

There is a reduction in mainly collagen type III, but also type I to some degree, in the skin of post-menopausal women compared to pre-menopausal women, resulting in a decrease in the ratio of type III/type I collagen within the dermis that is associated with estrogen deficiency (Affinito et al., 1999[2]; Horng et al., 2017[62]). When applied locally to the skin of post-menopausal women, estradiol significantly increases the production of hydroxyproline, reflecting elevated collagen synthesis in the dermis (Albright et al., 1941[3]; Affinito et al., 1999[2]; Sator et al., 2001[120]; Horng et al., 2017[62]). Indeed, topical estrogen improves the external facial appearance of post-menopausal women by reducing skin sagging and wrinkling (Schmidt et al., 1994[123]). Not only topical but also systemic estrogen supplementation conserves skin thickness by promoting dermal collagen deposition in post-menopausal women (Savvas et al., 1993[122]; Sauerbronn et al., 2000[121]). 

It has also been reported that estrogen replacement therapy can improve skin elasticity by 5 % per year (Brincat et al., 1987[27]). In line with this finding, topical estrogen supplementation improves the elasticity of ECM fibres in the dermis (Albright et al., 1941[3]; Sator et al., 2001[120]). Topical estrogen ointments notably increase the number and thickness of elastin fibres in the ECM, with histological examination demonstrating improved orientation and reduced fibre fragmentation in the dermis (Punnonen et al., 1987[112]). Estrogen also promotes the synthesis of glycosaminoglycans in the ECM, restoring skin turgor and moisture levels (Brincat, 2000[26]). 

Topical estrogen application enhances stratum corneum barrier function of skin in post-menopausal women and increases the rate of mitosis and turnover of epidermal cells (Stumpf et al., 1974[139]). Estrogen also enhances the vascularization of dermis and in terms of skin appendages, estrogen extends the life cycle of human hair follicles but retards hair growth and sebum secretion by sebaceous glands (Stumpf et al., 1974[139]).

In summary, the age-related fall in the levels of estrogen detrimentally affects the maintenance and turnover of intact skin, whilst estrogen supplementation reverses these effects in the elderly by stimulating keratinocyte proliferation, increasing ECM deposition and quality, and enhancing skin turgor and moisture retention.

## Estrogen and Wound Healing

The influence of estrogen on wound healing was first studied in animals in 1947 (Sjövall, 1947[132]; Horng et al., 2017[62]) and then in humans in 1953 (Sjöstedt, 1953[131]; Horng et al., 2017[62]). Subsequently, there has been an accumulating body of evidence supporting estrogen as a global regulator of wound healing (Brincat et al., 1987[27]; Varila et al., 1995[146]; Affinito et al., 1999[2]; Sauerbronn et al., 2000[121]; Mills et al., 2005[89]; Hardman and Ashcroft, 2008[56]; Brufani et al., 2009[28]; Lee et al., 2013[76]; Midgley et al., 2016[87]; Mukai et al., 2016[96]; Chenu et al., 2017[34]; Leblanc et al., 2017[75]; Horng et al., 2017[62]; Pepe et al., 2017[107]; Wilkinson and Hardman, 2017[154]). 

Research has demonstrated the key role of sex-steroid hormones in inflammation and the wound healing process (Guo and DiPietro, 2010[54]; Gilliver et al., 2007[50]). Estrogen has protective, anti-inflammatory properties in several tissues (Straub, 2007[138]). Estrogen has also been reported to stimulate wound repair processes such as re-epithelialization and ECM production independently from its anti-inflammatory effects in elderly subjects of both genders (Ashcroft et al., 1997[8]). HRT-treated post-menopausal women heal acute wounds faster than their age-matched control counterparts, who have taken no estrogen supplementation (Ashcroft et al., 1997[8]). Other reports indicate that topical estrogen supplementation enhances wound healing in elderly male and female patients, connected with a reduced inflammatory response (Ashcroft et al., 1997[8], 1999[9]).

Variances in the human immune system between male and female subjects have been identified in several epidemiological and medical studies (McGowan et al., 1975[85]; Bone, 1992[23]), with evidence indicating that women have a superior immune system compared to men (Gulshan et al., 1990[53]; Wichmann et al., 1996[153]). Other experiments have indicated that estrogen has immune-enhancing properties during stress, including increased resistance to several pathogenic infections (Yamamoto, 1999[157]).

Since systemic and peripheral estrogens decline with age, it is suggested that estrogen deprivation in the elderly could increase the propensity for chronic wounds. Margolis et al. (2002[82]) performed a case-cohort study to investigate the protective effects of estrogen against chronic wounds. Patients aged oved 65 years receiving HRT treatment were shown to be 30-40 % less likely to develop a venous leg ulcer than age-matched patients lacking HRT supplementation (Margolis et al., 2002[82]).

Chronic wounds are characterized by an excessive and chronic prolonged inflammation. High levels of inflammatory mediators, including tumor necrosis factor alpha (TNF-α), interleukin-1 beta (IL-1β), IL-6, IGF-1 and matrix metalloproteinases (MMPs), that are present in chronic wound exudate (Ashcroft et al., 1997[8], 1999[9]) are downregulated via the action of estrogen (Vural et al., 2006[148]; Straub, 2007[138]; Wira et al., 2015[155]). In particular, TNF-α is elevated in humans that are predisposed to chronic wounds and has been identified as a therapeutic target for impaired wound healing in the elderly (Ashcroft et al., 2012[13]). Both systemic and topical estrogen treatments enhance wound healing in elderly men and women by stimulating re-epithelialization, angiogenesis, matrix deposition and wound contraction whilst dampening the inflammatory response and expression of pro-inflammatory cytokines and proteolytic mediators (Ashcroft et al., 1997[8]; Ashcroft and Ashworth, 2003[7]; Thornton, 2013[145]; Archer, 2012[5]; Stevenson and Thornton, 2007[136]).

### Effect of estrogen on the inflammatory phase of wound healing

It is commonly known that age-related impaired healing is associated with an excessive and prolonged inflammatory response, linked with increased but delayed inflammatory cell recruitment, and increased secretion of pro-inflammatory cytokines such as TNF-α (Ralston et al., 1990[115]; Pottratz et al., 1994[111]). Moreover, TNF-α is elevated in elderly patients with venous ulcers compared to age-matched healthy controls, with the highest levels of TNF-α typically found in patients carrying polymorphisms of the promoter region of the ER-β gene that predispose to venous ulceration (Ashworth et al., 2008[15]).

Recent research has indicated that chronic wounds are associated with elevated levels of elastase and MMPs, which are released by macrophages, keratinocytes and fibroblasts, and linked with excessive tissue destruction (Wysocki et al., 1993[156]). Estrogen has been described to control and dampen the early inflammatory response during acute wound healing by inhibiting neutrophil infiltration to the wound via a reduction in the expression of cell adhesion molecules (Ashcroft et al., 1999[9]; Sproston et al., 2018[134]). Furthermore, estrogen increases the oxidative metabolism of neutrophils, suggesting estrogen deprivation could lead to diminished phagocytic capability of neutrophils, an increased risk of infection and a postponement in healing (Magnusson and Einarsson, 1990[81]). Estrogen has been shown to have a direct influence on monocytes and macrophages, due to their possession of both nuclear and membrane-bound estrogen receptor (ER) proteins (Weusten et al., 1986[151]; Suenaga et al., 1996[141], 1998[140]). In addition, 17β-estradiol has been reported to reverse the substantial delay in cutaneous murine wound healing induced by bacterial lipopolysaccharide (Crompton et al., 2016[38]). 

Increased levels of epidermal pro-matrix metalloproteinase-2 (pro-MMP-2) have been observed in intact aging skin and is immediately activated following cutaneous injury, explaining the reported rise in MMP-2 and ECM degeneration observed in the wounds of the elderly (Ashcroft et al., 1997[12]). In addition, research suggests that estrogen deficiency inhibits the differentiation of monocytes into tissue macrophages during the inflammatory phase of wound healing, leading to an increase in protease expression (Calvin et al., 1998[32]). Estrogen decreases tissue-damaging protease levels, including elastase and MMP secretion, leading to an overall increase in the content of collagen and fibronectin in the dermis (Ashcroft et al., 1999[9]).

In skin, the anti-inflammatory effect of estrogen is predominantly mediated through inhibition of the pro-inflammatory cytokine, macrophage migration inhibitory factor (MIF) (Hardman et al., 2005[57]). Macrophage migration inhibitory factor (MIF) has been identified as a global regulator of wound healing mediated by estrogen and released by monocytes, macrophages, neutrophils, endothelial cells and keratinocytes (Hardman et al., 2005[57]; Emmerson et al., 2009[44]). Ashcroft et al. (2003[11]) reported that mice with estrogen deficiency have higher MIF levels, resulting in an elevated inflammatory response and delayed wound healing, whereas MIF null-mice displayed enhanced wound healing, with lower inflammation and greater matrix formation. Estrogen downregulates MIF expression leading to a decline in inflammation, enhanced matrix deposition, increased re-epithelialization and an overall accelerated wound repair (Hardman et al., 2005[57]).

### Effect of estrogen on the proliferative phase of wound healing

Age-related impaired healing is linked with reduced growth factor expression, reduced keratinocyte proliferation and increased response to inhibitory cytokines, causing a delayed re-epithelialization *in vivo* (Butcher and Klingsberg, 1963[30]; Rattan and Derventzi, 1991[116]; Holt et al., 1992[61]). Estrogen enhances the mitogenesis of keratinocytes and increases re-epithelialization in post-menopausal women (Ashcroft et al., 1997[8]). It has been reported that the rate of wound re-epithelialization of post-menopausal women treated with HRT for more than 3 months was similar to levels of re-epithelialization in pre-menopausal females, whereas a non-HRT post-menopausal group showed diminished re-epithelialization. This improved re-epithelialization following estrogen supplementation is due to increased proliferation of epidermal keratinocytes (Raja et al., 2007[114]). 

In addition to its effect on epithelial migration and proliferation, estrogen indirectly effects matrix deposition by mesenchymal cells. Various *in vivo *animal studies report that estrogen increases fibroblast infiltration and collagen deposition. In contrast, a small number of studies report a decrease in fibroblast infiltration and collagen deposition following treatment with estrogen in mice (Lundgren, 1973[80]; Pallin et al., 1975[104]). A possible explanation for these contradictions include differences in the wound models, hormone concentrations and intervals of administration used. Furthermore, the duration of estrogen insufficiency results in distinct effects on several healing parameters; for instance, wound contraction becomes reduced after 4 months of estrogen deprivation whereas matrix deposition becomes reduced after only 1 month (Calvin et al., 1998[31]). In humans, topical estrogen supplementation in elderly men and women results in reduced wound size via stimulation of wound contraction (Ashcroft et al., 1999[9]). Estrogen promotes PDGF expression by monocytes and macrophages (Mendelsohn and Karas, 1999[86]), leading to mitogenesis and chemotaxis of fibroblasts and a subsequent increase in wound contraction and ECM deposition (Seppä et al., 1982[126]). Estrogen also enhances the secretion of TGF-β1 by dermal fibroblasts *in vivo* (Ashcroft et al., 1997[8], 1999[9]), resulting in enhanced formation of ECM, particularly collagen deposition (Ashcroft and Ashworth, 2003[7]). 

Estrogen promotes angiogenesis, leading to increased granulation tissue (Iyer et al., 2012[64]) through a direct stimulation of endothelial cells (Rubanyi et al., 2002[118]). Estrogen modulates the synthesis of IL-1 by tissue macrophages, a key protein implicated in the creation of a new granulation tissue (Hu et al., 1988[63]). Estrogen increases endothelial cell attachment to laminin, fibronectin and collagens I and IV *in vitro*. In addition, estrogen enhances the creation of capillary-like structures by endothelial cells, when positioned on a reconstructed basement membrane (Morales et al., 1995[92]). Paradoxically, other *in vitro* studies report a reduction in vascularity following stimulation with estrogen (Nyman, 1971[102]; Lundgren, 1973[80]). The precise effect of estrogen on angiogenesis remains unknown, and additional investigations are needed to define the impact of estrogen on vascularzsation in acute and impaired wound healing.

In summary, despite some contradictions in the literature, estrogen appears on balance to enhance most tissue formation occurring in the proliferative phase of wound healing, particularly re-epitheliazation and ECM formation.

### Effect of estrogen on the remodeling phase of wound healing

The age-related decline in estrogen levels causes a decrease in wound collagen and fibronectin *in vivo*. This has been associated with elevated levels of inflammatory cell-derived elastase, MMP-2 and MMP-9 (Herrick et al., 1997[59]; Ashcroft et al., 1997[12]). Estrogen supplementation reverses the degradation of ECM by inhibiting the synthesis of wound proteases such as MMPs during wound remodeling (Ashcroft and Ashworth, 2003[7]; Brincat, 2000[26]).

Topical estrogen supplementation increases the deposition of collagen during the remodeling phase of wound repair in elderly patients (Ashcroft et al., 1997[8], 1999[9]). Previous animal studies report that 17β-estradiol increases the production of tissue inhibitor of metalloproteinases (TIMPs) by rabbit uterine fibroblasts, but reduces the production of pro-collagenase and pro-stromelysin (Sato et al., 1991[119]). Another *in vivo* study reports that topical estrogen treatment increases collagen deposition in elderly males and females after 7 and 80 days post-injury (Ashcroft et al., 1999[9]). It was also noticed in other *in vivo* studies that matrix collagen deposition at 7 and 84 days post-wounding decreased in post-menopausal women lacking HRT treatment. In contrast, post-menopausal females who took HRT for more than 3 months had similar levels of matrix collagen deposition and wound remodeling as younger pre-menopausal females (Ashcroft et al., 1997b, 1999[9]).

Estrogen stimulates the expression of TGF-β1 *in vivo*. This results in improving collagen deposition in the dermis (Ashcroft et al., 1997[8]). Reports suggest a decreased wound collagen deposition associated with MMP-mediated collagenolysis in ovarectomized rats (Pirila et al., 2001[110]). These effects were reversed by estrogen replacement, implicating estrogen as a pivotal mediator involved in shifting the balance from matrix degradation to matrix synthesis (Pirila et al., 2001[110]). Interestingly, an *in vivo* study indicated that the quality of mature tissue scars was greater in post-menopausal women in comparison with pre-menopausal women. This suggests that estrogen enhances wound repair at the expense of scar quality (Ashcroft et al., 1997[8]).

## Future Perspectives for Estrogen Therapies

Although many known effects of estrogen on wound healing have been established in the past two decades, fewer recent developments have been made and there remain substantial areas for further investigation. It has been established that estrogen plays a fundamental beneficial role in skin maintenance and acute wound healing processes. Moreover, the systemic and peripheral decline in estrogen with increasing age suggests estrogen deprivation could be linked with chronic wounds in the elderly. However, systemic estrogen replacement therapy is an unfocused, biological sledgehammer rather than a targeted treatment strategy. Although estrogen is protective against photoaging, an extrinsic aging process that correlates with higher mortality rates from skin cancers in men than women (Weinstock, 1994[150]; Miller and Neil, 1997[88]), unopposed systemic estrogen replacement therapy is a risk factor for breast and endometrial cancer development (Nuttall et al., 2001[101]), thereby restricting its exploitation in clinical practice. The widespread distribution of estrogen-responsive tissues exposes non-target cells to the potential hyper-proliferative and neoplastic effects of systemic estrogen therapies, suggesting either local estrogen or targeted therapies are needed. Interestingly, studies performed *in vitro* have shown that ER-β is the dominant partner in heterodimers, resulting in an ER-β-predominant effect with repressed ER-α transcriptional activity (Pettersson and Gustafsson, 2001[108]). Thus, by modulating ER-α-mediated gene transcription, ER-β may decrease the overall cellular sensitivity to estrogen and provide protection against the hyper-proliferative and neoplastic effects of ER-α (Rollerova and Urbancikova, 2000[117]). Thus, a clear understanding of tissue-specific regulation of ER expression and downstream cellular and molecular mechanisms of estrogen action might enable controlled manipulation of estrogen signaling pathways during wound repair, potentially leading to the development of more targeted therapies with fewer side effects on non-target tissues.

Selective estrogen receptor modulators (SERMs) are ER-interacting molecules that have the ability to bind ER proteins and act as agonists in some tissues whilst acting as antagonists in different tissues (Brzozowski et al., 1997[29]; Cho and Nuttall, 2001[35]). SERMs have been used clinically to promote the beneficial effects of estrogen in target tissues whilst reducing the detrimental effects of estrogen (e.g. increased risk of breast cancer) in non-target tissues (Mirkin and Pickar, 2015[90]). Tamoxifen, raloxifene and the dietary phytoestrogen genistein are the most frequently documented SERMs in the literature. They are known to have estrogenic effects in numerous peripheral tissues, but are anti-estrogenic in the breast tissue and are therefore used extensively in breast cancer research (Furr and Jordan, 1984[49]; Morris and Wakeling, 2002[93]; Park and Jordan, 2002[105]; Mirkin and Pickar, 2015[90]). Tamoxifen was discovered and reported by the Food and Drug Administration (FDA) in 1977 (Park and Jordan, 2002[105]; Jordan, 2006[66]; Mirkin and Pickar, 2015[90]; Quirke, 2017[113]). Tamoxifen binds to both ER proteins and its effect depends on the cell and tissue type, being anti-estrogenic in breast tissue and therefore commonly used to prevent and/or treat breast cancer in both post- and pre-menopausal females (Zidan et al., 2004[158]; Quirke, 2017[113]). Tamoxifen has also been reported to maintain the density of bone in rats and humans (Jordan et al., 1987[67]; Zidan et al., 2004[158]). However, it has multiple side effects and is frequently linked with endometrial cancer due to its estrogenic effects in the uterus (Kedar et al., 1994[68]).

There have been some investigations on the effect of SERMs on skin and wound healing processes. Tamoxifen and raloxifene have been shown to stimulate fibroblast proliferation *in vitro* (Stevenson et al., 2009[135]). While raloxifene improves skin elasticity and collagen deposition in post-menopausal females (Sumino et al., 2009[142]), genistein has been reported to improve the vascularization of the dermis and augment the loss of epidermal thickness typically observed in post-menopausal females (Moraes et al., 2009[91]). Another study on mice indicated genistein stimulates wound healing via synthesis of TGF-β1 (Marini et al., 2010[83]). Moreover, tamoxifen, raloxifene and genistein all significantly enhance wound healing in ovariectomized mice by stimulating re-epithelialization and dampening inflammation via activation of ER-β (Hardman et al., 2008[58]; Emmerson et al., 2010[43]). However, the use of existing SERMs have not yet been exploited in the treatment of chronic wound states. 

The repurposing of existing pharmaceutical drugs or the development of novel therapies that act as ER-specific ligands or exhibit tissue-specific estrogenic effects, delivered locally within specialized wound dressings may have potential clinical applications in the treatment of chronic wound states in the elderly. Understanding the differential effects on downstream gene transcription or repression in various tissue/cell types may help develop more focused treatments for impaired wounds that can mediate specific estrogen-responsive signaling pathways in injured tissues whilst reducing unwanted side effects in non-target tissues.

## Conclusion

The literature indicates estrogen deficiency is a central paradigm of age-related impaired wound healing in both genders, with topical and systemic estrogen replacement reversing the detrimental effects of aging on both wound repair and skin maintenance. There is growing evidence indicating estrogen deprivation may also contribute to the development of chronic wounds in the elderly but further research is needed in this area. Interestingly, although the beneficial effects of estrogen on wound repair have been widely explored, the development of estrogen-based treatments to promote healing has failed to gain traction to date, most likely due to undesired cellular activity (including hyper-proliferative/neoplastic effects) in non-target tissues. However, a rekindled interest may be stimulated by prospects of developing targeted therapeutic strategies that might promote healing through selective activation of estrogen-responsive signaling pathways in regenerating peripheral tissues, whilst leaving non-target tissues largely unaffected.

## Conflict of interest

The authors declare no conflict of interest.

## Acknowledgement

The authors wish to acknowledge that this review article includes some aspects of the introductory chapters of the PhD thesis submitted by the lead author (Mohamed El Mohtadi).
